# Bacterial Response to the Surface Aging of PLA Matrices Loaded with Active Compounds

**DOI:** 10.3390/polym14224976

**Published:** 2022-11-17

**Authors:** María Fernández-Grajera, Amparo María Gallardo-Moreno, Verónica Luque-Agudo, María Luisa González-Martín, Margarita Hierro-Oliva

**Affiliations:** 1 Department of Applied Physics, Faculty of Science, University of Extremadura, 06006 Badajoz, Spain; 2 University Institute of Extremadura Sanity Research (INUBE), 06006 Badajoz, Spain; 3 Networking Research Center on Bioengineering, Biomaterials and Nanomedicine (CIBER-BBN), 06006 Badajoz, Spain; 4 Department of Natural Systems and Resources, E.T.S.I. of Forestry, Forestry and Natural Environment, Polytechnic University of Madrid, 28040 Madrid, Spain

**Keywords:** biomaterial, polylactic acid, surface characterization, surface aging, *Staphylococcus epidermidis*, antimicrobial compounds, magnesium, cetyltrimethylammonium bromide

## Abstract

The use of active components in biomaterials improves the properties of existing ones and makes it possible to obtain new devices with antibacterial properties that prevent infections after implantation, thus guaranteeing the success of the implant. In this work, cetyltrimethylammonium bromide (CTAB) and magnesium particles were incorporated into polylactic acid (PLA) films to assess the extent to which progressive aging of the new surfaces resists bacterial colonization processes. For this purpose, the films’ surface was characterized by contact angle measurements, ToF-SIMS and AFM, and adhesion, viability and biofilm growth of *Staphylococcus epidermidis* bacteria on these films were also evaluated. The results show that the inclusion of Mg and CTAB in PLA films changes their surface properties both before and after aging and also modifies bacterial adhesion on the polymer. Complete bactericidal activity is exhibited on non-degraded films and films with CTAB. This antibacterial behavior is maintained after degradation for three months in the case of films containing a higher amount of CTAB.

## 1. Introduction

The design and manufacture of new biomaterials for implant purposes is a field in constant development and has grown exponentially in recent years [1]. Parallel to this important progress is the inevitable increase of resistant microorganisms, which causes serious problems of infections in biomaterials used as implants [2]. Antibacterial treatment, once the infection has spread on these devices, is ineffective, since this procedure acts mainly on planktonic bacteria and not on those that are embedded and protected within a biofilm, a network-like structure unequivocally associated with infection [3,4,5]. The only way to solve the problem of implant infections is to replace the infected device by a new one, with the consequent health risks and economic implications for health systems [6].

One of the opportunistic bacteria present in a large number of nosocomial infections is *Staphylococcus epidermidis* [7,8]. These bacteria can colonize the surface of a biomaterial some time after implantation, due to the dissemination of bacteria from an active focus of infection, or during the implantation process, as a result of failures in the aseptic protocol [9], which means that the implant is already contaminated when it enters the patient [10,11].

For this reason, the development of new biomaterials and the improvement of existing ones to achieve devices with new antibacterial properties to prevent infections after implantation, and thus ensure the success of the implant, is a challenge [12]. In this context, biodegradable implants are ideal for preventing the development of latent infections since, once living tissue is restored, they disappear from the human body.

One of the biodegradable materials with lowest toxicity and highest sustainability and biocompatibility is polylactic acid (PLA). As with other polyesters, the polymeric structure that makes up PLA-based materials also has the advantage that it can be doped with different compounds to improve both the mechanical properties of the polymer and its response to bacterial colonization [13].

PLA’s weaknesses in mechanical properties are solved by including magnesium particles in the polymer matrix [14,15]. This metal, besides being biocompatible and having antibacterial activity, improves the Young’s modulus of PLA and, although it might seem unsuitable due to its corrosiveness and the generation of hydroxide ions, this is compensated with the acidification of the medium while PLA degradation occurs [11,16,17,18].

In addition to magnesium, some metal ions or atoms, such as silver [19,20], zinc [20] or copper [20,21,22] are also used to give PLA antibacterial properties. In this respect, the bibliography illustrates the use of other dopants of the matrix [13] by reinforcing or by impregnating it [23], such as antibiotics [24,25], surfactants [26,27,28], nanoparticles [29,30,31] and natural biological materials [32,33]. 

Cetyltrimethylammonium bromide (CTAB) is a cationic surfactant which has shown a double benefit in that it is able to reduce biofilm formation [26,27,28] and to improve the dispersion of magnesium particles within PLA matrices [34].

Unavoidably, any dopant introduced into the matrix can alter not only the physical properties of the bulk but also the surface properties. For example, the hydrophilicity of PLA can be improved by including CTAB on its surface [34]. The surface properties regulate the interaction between the implant and the surrounding physiological environment. Thus, the hydrophobicity of the material, its surface free energy or its topography are key in determining whether bacterial adhesion and proliferation, which is undesirable, and the formation of a protein layer that facilitates cell adhesion and proliferation, which is desirable, will occur [11,35]. Although it is difficult to find a general rule that describes the degree of involvement of each surface property in the bacterial colonization process, recently, Pahlevanzadeh et al. have indicated that hydrophilic, hydrated and non-charged surfaces have the lowest bacterial adhesion capacity [1]. This does not mean that hydrophobic materials are not suitable for medical use, since the final result, in terms of bacterial adhesion, is dictated not only by the overall surface properties of the material but also by the surface properties of the microbial. With regard to topography, its influence on bacterial adhesion processes is related to the shape, size and arrangement of surface structures. In general, in porous surfaces, if the pore size is smaller than the size of the bacteria, the number of bacteria adhering to the surface will be low [36]. On the other hand, certain surface structures, both random and ordered, do not promote bacterial adhesion [37,38,39]. Recently, it has been reported that porous and rough surfaces favor bacterial interaction and adhesion [1]. Furthermore, Tebbs et al. also reported that reduced roughness of biomedical implants results in less bacterial adhesion than in implants with irregular surfaces [40].

The passing of time can significantly change the surface of a material. This surface aging is especially important in biomaterials inserted inside the human body and, even more so in the case of biodegradable materials such as PLA. Therefore, the in vitro study of the surface properties should be carried out both on the recently prepared material and after an aging-degradation process that approximates in vivo conditions, taking into account that physiological fluids contain salts and other compounds that can chemically attack the surface of the implant and so its surface properties can be significantly altered [23]. It is therefore reasonable to assume that there may be a time dependence between the surface properties of a material and its response to bacterial colonization and growth [11]. A material that is initially smooth and not prone to bacterial adhesion may become rough after degradation, thus favoring adhesion. The process can be even more complex when the materials are loaded with active compounds: the degradation of such biomaterials assists in the progressive release of antibacterial dopants from the matrix, altering the response of the biomaterial to bacterial and cell adhesion [41].

In this scenario, the antibacterial response of the PLA surface, fabricated without and with active components, before and after being degraded for different periods of time, was evaluated. To this end, PLA films doped with magnesium particles and different amounts of CTAB were prepared, and adhesion, viability and biofilm growth of *S. epidermidis* bacteria on these films before and after degradation in PBS for 1 and 3 months was evaluated. Our study also includes the analysis of the surface properties (hydrophobicity, surface free energy, surface chemical composition and topography) of the samples before and after degradation, in order to check whether aging of the material affects the bacterial surface coating.

## 2. Materials and Methods

### 2.1. Materials and Chemical Reagents

PLA particles PLA2003D, with a D-isomer content of 4.25%, were purchased from NatureWorks LLC (Blair, NE, USA). Chloroform and CTAB were purchased from Sigma-Aldrich (Merck, Darmstadt, Germany) and magnesium particles (≤50 μm) are from Nitroparis (Castellón, Spain). For contact angle measurements, deionized water (Milli-Q Integral 5 System, Merck, Darmstadt, Germany) and diiodomethane (Fluka, Thermo Fisher Scientific, Waltham, MA, USA) were used. For bacterial assays, Tryptic Soy Broth (TSB) medium was purchased from BBL, Becton Dickinson and Company (Sparks, Franklin Lakes, NJ, USA) and viable biofilms were quantified using BacTiter-Glo™ Microbial Cell Viability Assay (Promega Corporation, Madison, WI, USA).

### 2.2. Preparation of Films

**Figure 1:**
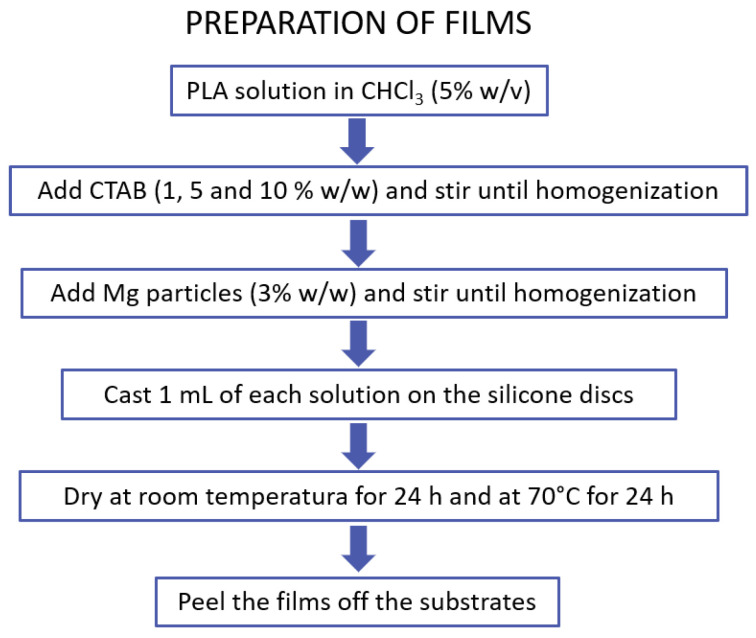
Preparation of films containing CTAB and magnesium particles.

PLA films were prepared on silicone supports, with different amounts of CTAB and magnesium particles, as detailed in our previous work [34]. Briefly, the silicone discs to be used as supports were first cleaned with absolute ethanol and then dried with a flow of N2. Thereafter, the PLA particles were dissolved in chloroform (5% *w*/*v*) using a rotary stirrer (JP Selecta, Barcelona, Spain). Different amounts of CTAB (0, 1 and 5% *w*/*w* relative to PLA) were then added and the solutions were homogenized by stirring. To prepare the magnesium-containing solutions, magnesium particles (≤50 μm) were added and stirred to a final concentration of 3% *w*/*w* relative to PLA. After preparing all the solutions, 1 mL of each solution was deposited on the silicone substrates and dried at room temperature for 24 h. The films were then dried in an oven at 70 °C for 24 h, in order to completely remove any remaining solvent. These films were peeled off from the silicone after drying. The process is depicted in the scheme shown in Figure 1.

### 2.3. In Vitro Degradation

The films were degraded by immersing them vertically in 100 mL of phosphate buffered saline (PBS) at 37 °C with the help of small hanger hooks. Two degradation times were selected: 1 and 3 months. After these times, the films were dried with a flow of N_2_ and stored in a desiccator until use. Because of all the variables involved in each sample and to better follow the results, the notation used is the following: X/Y_Z_, where X refers to the amount of magnesium added (0 or 3% *w*/*w*), Y the amount of surfactant added (0, 1 or 5% *w*/*w*) and _Z_ the number of months of degradation (1 or 3).

### 2.4. Contact Angle Measurements and Determination of Surface Tension

Contact angle measurements of deionized water and diiodomethane were carried out with a Drop Shape Analyzer—DSA100E (Krüss, Hamburg, Germany) by the sessile drop method at room temperature under ambient conditions. Drops of 5 μL of deionized water and 2 μL of diiodomethane were placed at different positions on the surface of the samples and recorded 15 s after the complete deposition of each drop. The results of the contact angles were averaged values of at least three independent films for each of the studied conditions. The measured contact angles of the two liquids were used to calculate the surface tension and components of the samples using the Owens–Wendt–Kaelble (OWK) approach [42,43].

### 2.5. Surface Chemical Composition

Surface samples analyses by time of flight secondary ion mass spectrometry (ToF-SIMS) were performed with a Tof-SIMS^5^ (ION TOF, Münster, Germany) using a Bi_3_^2+^ as primary gun, operating at 25 keV under high vacuum conditions (2.4 × 10^−9^ mbar). Negative spectra were recorded with a total ion dose above 10^12^ ions/cm^2^. For each mass spectrum, a spot size of 200 × 200 μm^2^ with a resolution of 128 × 128 pixels was used. The mode of operation used was HC-BUNCHED with lateral resolution of 1 µm and a pulsed low energy electron flood gun was also used for charge neutralization. Spectra were collected with a cycle time of 100 µs. 

### 2.6. Surface Topography Characterization

Surface topography of the PLA films was evaluated using an atomic force microscope (AFM) (Agilent AFM 5500, Agilent Technologies, California, CA, USA) operating at room temperature and in contact mode. Rectangular cantilevers (FMV-PT, Bruker, Billerica, MA, USA) were used with a nominal spring constant of 75 kHz and a force constant of 2.8 N/m. Images were taken for two scanning lengths (50 × 50 μm^2^ and 25 × 25 μm^2^) and in each scanning area, topographical, deflection and friction images were acquired. Experiments were performed in triplicate, studying three different films for each condition. Roughness is a scale-dependent function and therefore all comparisons were made with the mean roughness parameter (Sa) always obtained from 50 × 50 μm^2^ images. Results are presented as mean values and standard deviations.

### 2.7. Bacterial Strains and Culture

*Staphylococcus epidermidis* ATCC 35983 (ATCC, American Type Collection Culture) was the bacterium used to perform the biofilm assays on PLA films. The pathogen was taken from stock at −80 °C in porous beads (Microbank, ProLab Diagnostics, Richmond Hill, ON, Canada) and cultured on blood agar plates (OXOID Ltd., Basingstoke, Hampshire, UK) for 24 h at 37 °C. To grow the bacteria in liquid medium, a colony was inoculated in Tryptic Soy Broth (TSB) medium overnight at 37 °C.

#### 2.7.1. Adhesion Experiments

Once the *Staphylococcus epidermidis* culture reached the exponential phase, bacteria were centrifuged (5 min at 1000× *g*) and washed in phosphate buffered saline (PBS, pH 7.4) three times. Then, they were resuspended in PBS at a concentration of 3 × 10^8^ bacteria/mL. Films were fixed to a flat support with double side sticky tape and then 1 mL of the bacterial suspension was kept in contact with the samples, with the help of silicone adhesion chambers, for 4 h at 37 °C under orbital shaking. Quantification of adhesion, after removal of loosely bound bacteria, was carried out on an epifluorescence microscope (Leitz DIAPLAN, Wetzlar, Germany) by staining adherent microorganisms with the Live/Dead Baclight L-7012 kit (Invitrogen SA, Eugene, OR, USA). Bacteria were counted with NIS-Elements BR 4.10 software (Nikon Instruments Inc., Melville, NY, USA). Green-colored bacteria are considered to be alive and those colored in red are associated with damaged or dead bacteria. Experiments were performed in triplicate and with independent cultures to confirm reproducibility.

#### 2.7.2. Biofilms Formation

The protocol detailed in previous studies [18,44] was followed to analyze the formation of biofilm on PLA. Specifically, after growth, bacterial suspension was set at 82% of transmittance at 492 nm using a horizontal light spectrophotometer (Helios epsilon model, Thermo Spectronic, Thermo Fisher Scientific Inc., Cambridge, UK), and then was diluted 1/100 to obtain a concentration of 10^6^ CFU/mL. After that, PLA samples were placed in a 24-well plate and 1 mL of the bacterial suspension was deposited on the surface of each PLA film for 24 h at 37 °C with gentle shaking. After the incubation time, the supernatant was removed from each sample and carefully washed twice with TSB to remove unattached bacteria. Viable biofilm-forming bacteria were then quantified using BacTiter-Glo™ Microbial Cell Viability Assay. After 5 min in darkness and light agitation, the supernatant was transferred to a 96-well white polystyrene flat-bottom microtiter plate (Greiner bio-one, Frickenhausen, Germany) and the emitted light was quantified with a fluorescence microplate reader (FLx800; Bio-Tek Instruments, Inc., Winooski, VT, USA). Experiments were performed in triplicate and with independent cultures to confirm reproducibility.

### 2.8. Static Analysis

Results are presented as mean ± SD. Statistical analysis was carried out with R statistical software package (The R Foundation for Statistical Computing, www.r-project.org accessed on 10 September 2022—R Core Team, 2014). Data were analyzed using one-way analysis of variance (ANOVA) with a Tukey post-hoc test. All analyses were performed at a significance level of *p* < 0.05.

## 3. Results

### 3.1. Films’ Surface Characterization

#### 3.1.1. Contact Angle Measurements

**Figure 2:**
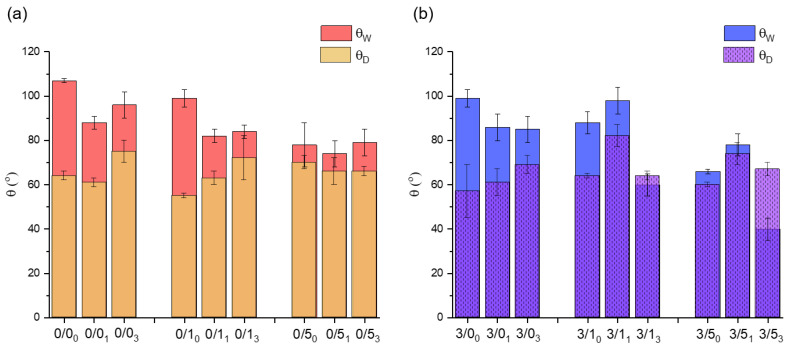
Contact angles of water (θ_W_) and diiodomethane (θ_D_) on films made of PLA, Mg particles and CTAB for non-degraded and degraded samples for 1 and 3 months. (**a**) Non-containing magnesium films; (**b**) magnesium-containing films. The texture of θ_D_ is included to differentiate more clearly between cases in which θ_D_ > θ_W_. All data are given as mean and standard deviation.

The water (θ_W_) and diiodomethane (θ_D_) contact angles of the prepared polymeric films are summarized in Figure 2. In order to make the results clearer, they are presented as a function of whether or not they contain magnesium and CTAB, and also as a function of degradation over time.

- Magnesium effect
  In most cases, the addition of 3% (*w*/*w*) magnesium particles to PLA films results in a decrease of θ_W_ (Figure 2). Nevertheless, the samples degraded for 1 month do not follow the described trend (3/1_1_ and 3/5_1_). On the other hand, no clear tendency can be deduced with respect to θ_D_, as in some cases, the value decreases with the addition of magnesium (0/0 vs. 3/0, whatever the degradation time and 0/5_0_ vs. 3/5_0_). In others cases, it increases slightly (0/1 vs. 3/1, except 0/1_3_ vs. 3/1_3_). Only for the case of samples degraded for 1 month is there a considerable increase in the value of the diiodomethane contact angle with Mg addition.
- CTAB effect
  With non-degraded films, regardless of whether the films contained magnesium particles or not, doping the films with CTAB results in a decrease in θ_W_, with the films being more hydrophilic the higher the CTAB content (Figure 2). Thus, there was a decrease of about 29° and about 33° in θ_W_ with respect to the control for films doped with 5% (*w*/*w*) CTAB, without and with magnesium, respectively: 0/5_0_ vs. 0/0_0_ and 3/5_0_ vs. 3/0_0_. The opposite trend was observed for θ_D_ values. In these cases, there was a slight increase with respect to the controls (0/0 and 3/0) as the CTAB content in the films increased (0/5_0_ vs. 0/0_0_ and 3/5_0_ vs. 3/0_0_), with the exception of the 0/1_0_ film, where θ_D_ decreased slightly.
  In the case of degraded films, the addition of CTAB also made the surfaces less hydrophobic on average, especially in films with 5% (*w*/*w*) CTAB.
- Degradation effect
  For magnesium-free films (Figure 2a), degradation of films immersed in PBS for 1 and 3 months resulted in a decrease of θW, compared to non-degraded films, in the cases of zero or a low amount of CTAB, i.e., 0/0 and 0/1. Moreover, this decrease in hydrophobicity was much more evident in the first 4 weeks than in the remaining 8 weeks. In the case of 0/5, the hydrophobicity of the films was similar before and after degradation. In addition, after 3 months degradation (0/03, 0/13 and 0/53), the average θ_W_ seemed to increase slightly with respect to the degraded films for 1 month.
  When magnesium was present in the films (Figure 2b), θ_W_ also decreased for samples degraded for 1 and 3 months, except for samples degraded for 1 month and containing 1% and 5% (*w*/*w*) CTAB. In this sense, the θ_W_ for the 3/1_1_ sample was higher than for the 3/1_0_ sample, and was also much higher than for the rest of the samples degraded for 1 month with different amounts of CTAB (3/0_1_ and 3/5_1_).
  In relation to the contact angle values of diiodomethane, degradation caused different trends depending on the presence or absence of magnesium and surfactant. In the samples without CTAB, 0/0 and 3/0, degradation after 1 month did not cause significant changes in θ_D_, but it did after 3 months, at which time the θ_D_ increased significantly with respect to the non-degraded samples.
  In the samples without Mg and with small amounts of CTAB (0/1, Figure 2a), the average value of θ_D_ seemed to rise with the degradation time, while when more CTAB was added (0/5), the values of the angles were very similar with time, as was the case with θ_W_.
  In the samples with Mg (Figure 2b), θ_D_, irrespective of the amount of CTAB, increased after 1 month of degradation, relative to the non-degraded samples, and equaled the original θ_D_ value after 3 months of degradation.

#### 3.1.2. Surface Tension

After applying the Owens–Wendt–Kaelble (OWK) approximation with the contact angle data of water and diiodomethane, the surface tension of the solid (γ_s_) and the dispersive (γ^d^) and non-dispersive (γ^nd^) components were obtained (Table 1).

- Magnesium effect
  Regardless of the sample selected, the total surface tension of the films was modulated by **γ**^d^ and **γ**^nd^ values. In the cases without Mg, the sum of both components gave **γ**_s_ values very similar to each other, taking into account experimental uncertainty. For the cases with Mg, there was more variability in γ_s_. The lowest value of 3/1_1_ and the highest value of 3/5_3_ are noteworthy, and in turn correspond to the highest value of θ_D_ and the lowest value of θ_W_, respectively.
  There were noticeable differences in the non-dispersive component, γ^nd^. In the case of non-degraded samples, magnesium-containing samples without or with small amounts of CTAB (3/0_0_ and 3/1_0_) had a low, but non-zero γ^nd^ value, which infers a certain polarity to the surface, while those non-containing magnesium (0/0_0_ and 0/1_0_) were non-polar (γ^nd^ = 0 mJ/m^2^).
  Table 1 also shows the free energy of interaction between two surfaces (ΔG_SWS_), which provides the degree of hydrophobicity of a surface when immersed in water. Taking this parameter into account, a surface is considered hydrophobic when ΔG_SWS_ < 0 mJ/m^2^ and hydrophilic when ΔG_SWS_ > 0 mJ/m^2^ [45]. Thus, the more negative this value is, the more hydrophobic the material. In general, the 0/Y_Z_ films have lower ΔG_SWS_ values than the 3/Y_Z_ films and therefore interaction with water is not favored in them, with the exception of 0/1_1_ vs. 3/1_1_.
- CTAB effect
  The addition of 1% *w*/*w* CTAB did not produce significant differences in most of the parameters presented in Table 1, bearing in mind the results uncertainty. An exception to this is the non-dispersive component, which increased in value when 1% *w*/*w* CTAB was added to PLA films, without Mg. Another exception occurred for sample 3/1_1_ where the decrease in the dispersive surface tension component provoked the described decrease in γ_s_ and the increase in ΔG_SWS_.
  However, in most of the cases, and irrespective of the starting value, an important increase of the non-dispersive component was observed when 5% *w*/*w* CTAB was added, and this was especially noticeable in magnesium-containing samples.
  As described above, all the films examined had a hydrophobic character (ΔG_SWS_ < 0 mJ/m^2^), but an appreciable decrease was noted as the amount of CTAB increased, especially when comparing 5% *w*/*w* CTAB with samples without CTAB.
- Degradation effect
  After degradation, higher values of the non-dispersive component seemed to be appreciated, although it is difficult to establish a time dependence. This was especially noticeable for the 3/Y_3_ (where Y is 0, 1 or 5) samples, whose value of the non-dispersive component is four times higher on average than for the non-degraded samples. These changes are reflected in the total surface tension of the solid, which, if taking into account the uncertainties associated with each value (as mentioned above), is generally higher for magnesium-containing films than for non-containing magnesium films, especially for the samples with the highest amount of CTAB.
  On comparing magnesium-containing samples before and after 3 months of degradation, the polarity of the samples increased by 350% for 3/1_3_ and 153% for 3/5_3_.
  Regarding ΔG_SWS_, a decrease in hydrophobic character was also observed after the samples were subjected to degradation, in particular for magnesium-containing samples. Again, it is worth noting the case of 3/5_3_ with the lowest hydrophobicity found, according to its maximum surface energy (γ_s_ = 58 ± 6 mJ/m^2^).

#### 3.1.3. Compositional Changes after Film Degradation

**Figure 3:**
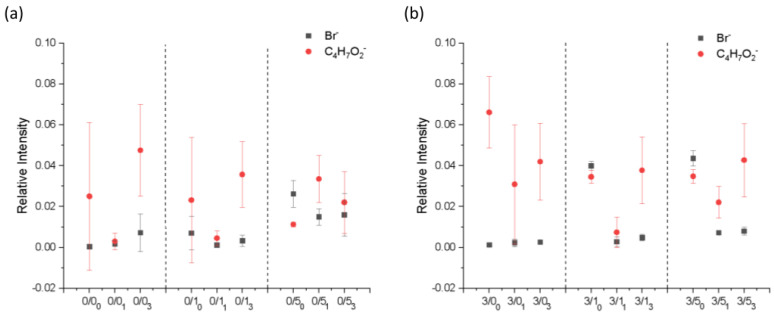
Relative intensities of Br^−^ and C_4_H_7_O_2_^−^ ions as a function of sample type for PLA films after degradation for 1 and 3 months. (**a**) PLA doped with CTAB; (**b**) PLA doped with magnesium and CTAB. All data are given as mean and standard deviation.

The surface composition of the films after degradation for 1 and 3 months was evaluated by ToF-SIMS surface mass spectra analysis. A similar analysis was performed for the non-degraded samples, published in a previous work carried out by our research group [34]. Data for non-degraded films have been included in the results for better comparison. Figure 3 shows the relative intensities of the characteristic ions of the surfactant (the bromide ion, Br^−^, was chosen) and the polymer (the C_4_H_7_O_2_^−^ fragment was selected) as a function of sample type. Both CTAB and PLA have hydrocarbon moieties in their structures, but the one considered for the analysis was unequivocally assigned to PLA and not to CTAB as it contains oxygen and no nitrogen.

- Magnesium effect
  In the case of non-degraded samples [34], the presence of Mg in PLA films made CTAB more significantly detectable on the surface of the samples, the change being more evident for lower concentrations of CTAB. The presence of magnesium in the films of PLA seemed to reduce the amount of surfactant on the surface of the degraded samples. This was more evident in samples with a higher amount of CTAB (0/5 vs. 3/5).
- CTAB effect
  As was the case for the non-degraded samples, in general, the bromide ion was barely detectable on the surface of films prepared with low concentrations of CTAB, and when the surfactant concentration increased, the presence of the surfactant became noticeable on the surface.
- Degradation effect
  Based on the results, after degradation, the amount of CTAB on the surface significantly decreased, regardless of whether the sample had been 1 month or 3 months under degradation.

#### 3.1.4. Topography of PLA Films

A recently published study carried out by our research group showed in great detail the topography that appears on PLA films depending on the amount of CTAB contained in the film, the substrate on which it was cast and whether or not it contained magnesium [41]. The present study also works with those films degraded for 1 month and adds a longer degradation time of 3 months. By this reason, the information presented in the present work will serve to complete the previous topographic analysis and, therefore, some previous results will be mentioned to better understand the current data.

**Figure 4:**
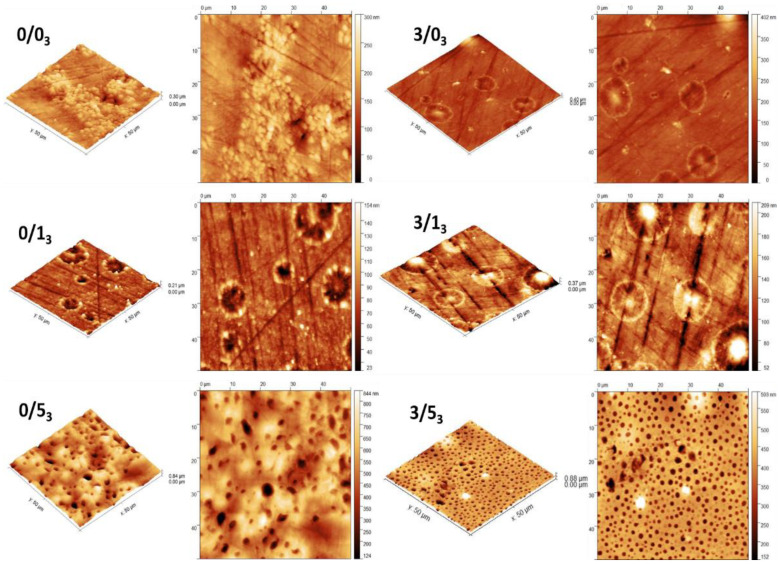
AFM images of films non-magnesium-containing and magnesium-containing and with CTAB after degradation for 3 months. For each kind of sample, left and right images are topographical and deflection images, respectively.

Figure 4 shows the new AFM images after 3 months degradation for samples without and with Mg and with the different amounts of CTAB.

- Magnesium effect
  We observed in the previous work [41] that the topography of films fabricated on silicone was not affected by the single addition of Mg, that it was affected when this Mg was combined with CTAB, or the films were subjected to degradation. This observation was also evidenced when comparing 0/5_3_ and 3/5_3_ where a more uniform hole-like distribution appeared in the presence of Mg. In the cases of 0/1_3_ vs. 3/1_3_, images were similar with no change associated to Mg.
- CTAB effect
  Samples without CTAB did not present holes on their surface, but the addition of 1% *w*/*w* CTAB provoked the appearance of small holes, without or with Mg, as described in the previous publication [41]. Moreover, the size of these microstructures increased with the CTAB content in the range of 1 to 5 µm [41]. In the cases under study (Figure 4), holes were only present in 0/5_3_ and 3/5_3_.
- Degradation effect
  Figure 4 shows the presence of holes on the surface of the films with the highest amount of CTAB after 3 months degradation (0/5_3_ and 3/5_3_) without or with Mg. However, for non-containing CTAB films (0/0_3_ and 3/0_3_) or with 1% (*w*/*w*) CTAB (0/1_3_ and 3/1_3_), circular-like deposited clusters appeared on the surface.
  Previous work [41] has shown that for non-containing-magnesium samples, the degradation after 1 month did not significantly affect the holes formed on the surface of the films when the amount of CTAB was higher than 5% (0/5), but in the case of samples with a lower amount of CTAB (0/1), these holes disappeared. Conversely, the presence of magnesium in the films degraded for 1 month seemed to affect the topography in the case of the high CTAB-content samples: some structures became wider and deeper than in the case of non-degraded film. In the case under study, after 3 months of degradation (Figure 4), the behavior was similar to 1 month: the surface of the films with low amounts of CTAB (0/1_3_ and 3/1_3_) did not show any holes after degradation, regardless of whether the films contained magnesium or not. For the samples with the highest amount of CTAB (0/5 and 3/5), those holes observed on the surface at 1 month degradation were still observed after 3 months of degradation, with the particularity that magnesium made the distribution of the structures more uniform, as we have already mentioned.
  Comparing these images with those already analyzed (0/5_3_ vs. 0/5_0_) [41], in the non-containing-magnesium samples with the highest amount of CTAB and degraded for 3 months (0/5_3_), there was a considerable increase in the width and depth of these holes, becoming, in the most extreme cases, 170% wider and 250% deeper on average (holes were on average 5 ± 0.6 μm wide and 500 ± 30 nm deep). On the other hand, for samples containing magnesium and the highest amount of CTAB (3/5_3_), there was no difference in the width and depth of the holes after 3 months of degradation compared to 1 month (holes were on average 1.9 ± 0.2 μm wide and 183 ± 40 nm deep).

### 3.2. Bacterial Adhesion and Viability of Adhered Bacteria

**Figure 5:**
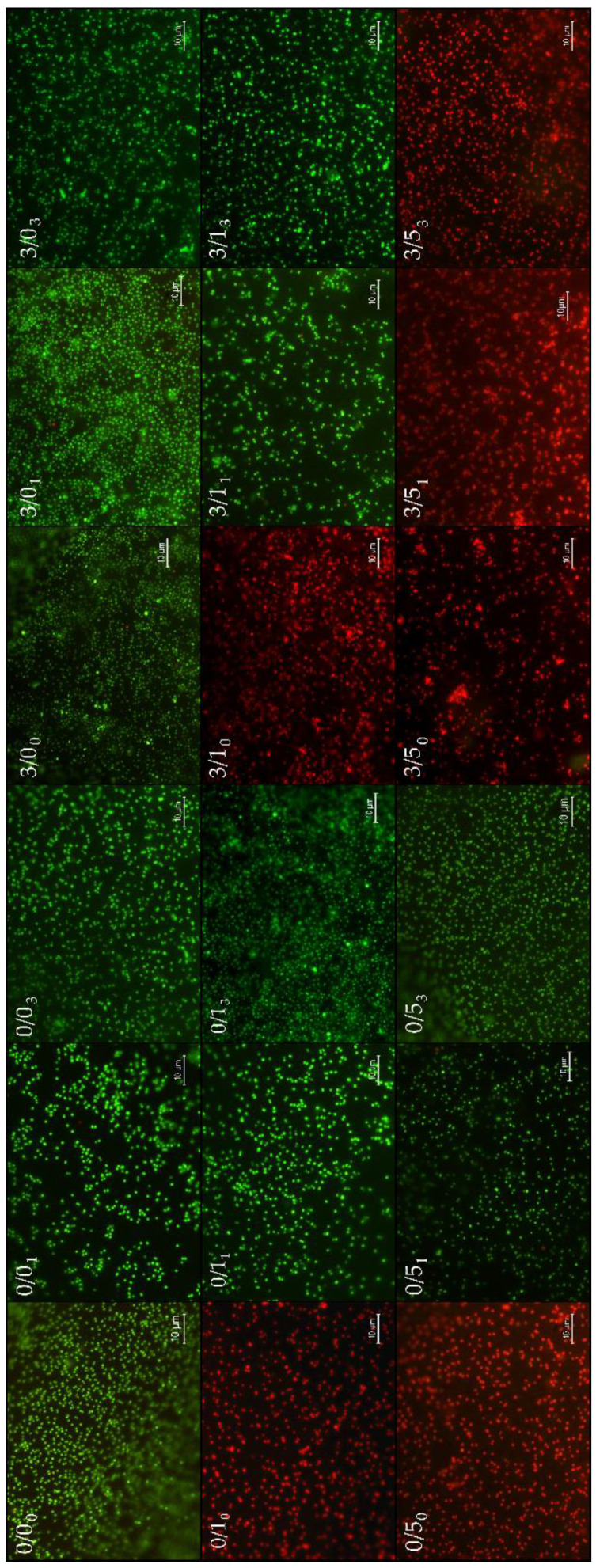
Representative fluorescence microscopy images of every PLA surfaces after bacterial adhesion. In the first row, we find the samples without CTAB from left to right 0/0_0_, 0/0_1_, 0/0_3_, 3/0_0_, 3/0_1_ y 3/0_0_. In the second row, the samples with 1%/*w*/*w*), the same from left to right 0/1_0_, 0/1_1_, 0/1_3_, 3/1_0_, 3/1_1_ y 3/1_3_. Then, in the last row are located the samples with 5% (*w*/*w*) CTAB from left to right 0/5_0_, 0/5_1_, 0/5_3_, 3/5_0_, 3/5_1_ y 3/5_3_. Green colored bacteria indicate that they are alive, and when bacteria show a red color they are damaged or not dead.

**Figure 6:**
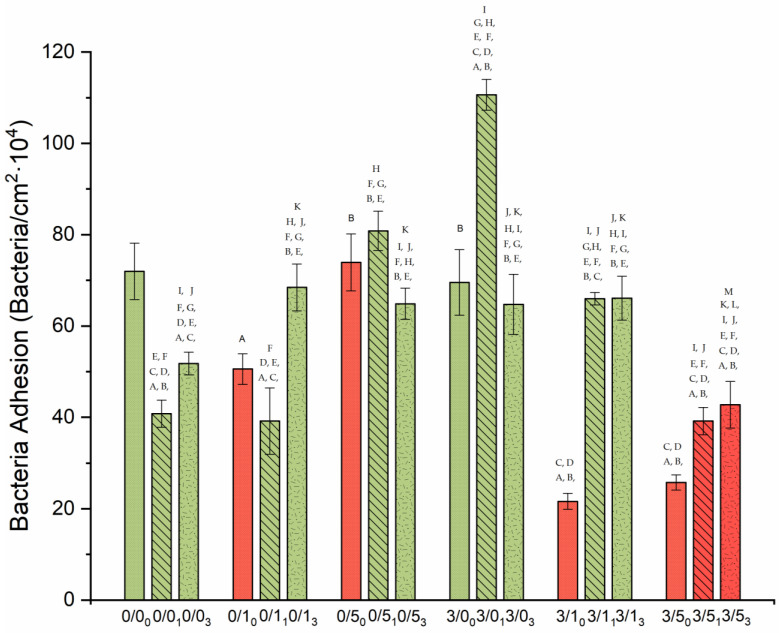
Bacterial density adhered to the surface of the PLA samples. The smooth bars are those given to the non-degraded samples, the diagonally hatched bars are for the samples degraded for one month, and lastly, the notched bars are for the samples degraded for 3 months. Likewise, the color given to each bar represents the viability of the adhered bacteria, green for the viable ones and red for the damaged ones. Statistical difference (*p* < 0.05) is marked with the different letters depending on which sample it is compared with. The letters ^A^ is used to 0/0_0_; ^B^ is used to 0/1_0_; ^C^ is used to 0/5_0_; ^D^ is used to 3/0_0_; ^E^ is used to 3/1_0_; ^F^ is used to 3/5_0_; ^G^ is used to 0/0_1_; ^H^ is used to 0/1_1_; ^I^ is used to 0/5_1_; ^J^ is used to 3/0_1_; ^K^ is used to 0/0_3_; ^L^ is used to 0/5_3_; ^M^ is used to 3/0_3_.

To have a visual perspective of the bacterial adhesion performance, Figure 5 presents a collage of the most representative images of each one of the surfaces, for short adhesion times (4 h). Figure 6 quantifies that adhesion process with two complementary information: number of adhered bacteria to the different films (read from the *Y*-axis) and viability of these adhered microorganisms inferred from the bar color, i.e., green (live) and red (damaged or dead).

- Magnesium effect
  The single addition of Mg to PLA films showed no significant change (*p*-value > 0.05) in bacterial adhesion (comparisons between 0/0_0_ and 3/0_0_). However, when this addition was made in the presence of CTAB, the number of adhered bacteria decreased significantly (*p*-value < 0.05), regardless of the amount of CTAB: comparisons between 0/1_0_ and 3/1_0_ (from 51 ± 2 × 10^4^ to 21 ± 1 × 10^4^ n.° bacteria/cm^2^) and 0/5_0_ and 3/5_0_ (from 70 ± 6 × 10^4^ to 26 ± 2 × 10^4^ n.° bacteria/cm^2^). Additionally, it is important to point out that bacteria adhered to 0/1_0_, 3/1_0_, 0/5_0_ and 3/5_0_ were damaged or dead due to the red-like color present after staining.
  When Mg was present in degraded samples, it was not possible to describe a general behavior. After 1 month, Mg addition caused a significantly increase (*p* < 0.05) in the number of adhered bacteria (0/0_1_ vs. 3/0_1_ and 0/1_1_ vs. 3/1_1_), except for those with 5% (*w*/*w*) CTAB, where there was a significant reduction (*p* < 0.05). In the case of degradation for 3 months, the adhesion was maintained, without and with Mg, within the experimental error (0/0_3_ vs. 3/0_3_ and 0/1_3_ vs. 3/1_3_), the behavior of the samples again being different with 5% (*w*/*w*) CTAB, which generated significantly less *p* < 0.05) adhesion with degradation.

- CTAB effect
  The presence of 1% (*w*/*w*) CTAB in the samples without and with Mg caused a significant decrease (*p* < 0.05) in bacterial adhesion: between 0/0_0_ and 0/1_0_ (from 72 ± 7 × 10^4^ to 50 ± 2 × 10^4^ bacteria/cm^2^) and between 3/0_0_ and 3/1_0_ (from 68 ± 6 × 10^4^ to 21 ± 1 × 10^4^ bacteria/cm^2^). In addition, as mentioned before, with these amounts of CTAB, the films always presented bactericidal activity in the adhered microorganisms.
  When films had high concentrations of CTAB (0/5_0_), the number of adhered bacteria was statistically equal to the “control” (0/0_0_) in the cases without Mg, but the bactericidal surface activity was, again, only associated to CTAB presence. If magnesium was present, in addition to the bactericidal effect associated with CTAB, adhesion was significantly reduced (*p* < 0.05) (compare 3/0_0_ with 3/5_0_: from 68 ± 6 × 10^4^ to 26 ± 2 × 10^4^ bacteria/cm^2^).
  Another comparison was made within samples with both amounts of CTAB. Figure 4 shows that in the cases without Mg (0/1_0_ and 0/5_0_), the higher the CTAB concentration, the greater the adhesion (*p* < 0.05). In the cases with Mg (3/1_0_ and 3/5_0_), the average adhesion values were very similar, although adhesion also seemed to increase with concentration. However, what is most noticeable in these compared cases is the bactericidal power of the surface.
  In the cases of degraded samples, the addition of 5% (*w*/*w*) surfactant always caused a significant increase (*p* < 0.05) in adhesion without Mg (0/0_1_ vs. 0/5_1_ and 0/0_3_ vs. 0/5_3_), whereas when Mg was present, it significantly decreased (*p* < 0.05) adhesion (3/0_1_ vs. 3/5_1_ and 3/0_3_ vs. 3/5_3_). In the case of 1% (*w*/*w*) CTAB, the coverage increased significantly *p* < 0.05, decreased or remained constant depending on the film additive and degradation time.
- Degradation effect
  Overall, degradation alters the bacterial surface coating, however, it is not possible to describe a generalized trend for all the systems studied. This means that the presence of magnesium and/or the presence of different amounts of CTAB are able to modulate bacterial adhesion and viability in different ways with time.
  Figure 6 shows the comparison with the non-degraded films, and that after 1 month degradation, the bacterial adhesion was significantly decreased (*p* < 0.05) in one case 0/0_1_ (41 ± 3 × 10^4^ bacteria/cm^2^), remained unchanged in 0/1_1_ and 0/5_1_ and increased (*p*-value < 0.05) in all Mg-containing films, 3/0_1_ (111 ± 4 × 10^4^ bacteria/cm^2^), 3/1_1_ (66 ± 1 × 10^4^ bacteria/cm^2^) and 3/5_1_ (39 ± 3 × 10^4^ bacteria/cm^2^). This degradation time was also crucial for the bactericidal effect initially shown in the 0/1_0_, 0/5_0_ and 3/1_0_ systems to disappear. The bactericidal capacity of the surfaces was only maintained after 1 month of degradation time for films with 5% (*w*/*w*) CTAB, i.e., 3/5_1_.
  After 3 months degradation, compared with non-degraded films, bacterial adhesion also decreased in the system 0/0_3_ (from 53 ± 2 × 10^4^ bacteria/cm^2^ to 72 ± 7 × 10^4^ bacteria/cm^2^), while in the case 0/1_3_, it increased (68 ± 5 × 10^4^ bacteria/cm^2^), but not significantly (*p*-value > 0.05). The film 0/5_3_ slightly decreased significantly (*p* < 0.05) the number of adhered bacteria, around 10 fewer bacteria/cm^2^. In the magnesium cases, for 3/0_3_, despite the large increase after 1 month of degradation, after 3 months of degradation, the bacterial coverage was similar to 3/0_0_. A different behavior was seen for 3/1 and 3/5, where 3/1_3_ and 3/5_3_ films had significantly (*p*-value < 0.05) more bacteria coverage than their non-degraded systems. Again, the bactericidal capacity of the 3/5 film surface even after 3 months degradation is noteworthy.
  In samples without Mg, there was an increase in bacterial coverage on the surface in 0/0 and 0/1 samples related to the significant increase (*p* < 0.05) in degradation time (0/0_1_ vs. 0/0_3_ and 0/1_1_ vs. 0/1_3_). However, in 0/5 films, degradation generated the opposite effect, where less degradation was associated with significantly higher (*p* < 0.05) bacterial adhesion (0/5_1_ vs. 0/5_3_). In the cases with Mg, for 3/1 and 3/5 films, a similar coverage was found after 1 and 3 months of degradation. In the case of sample 3/0, the longer the degradation time, the lower the bacterial adhesion.

### 3.3. Biofilms Formation

**Figure 7:**
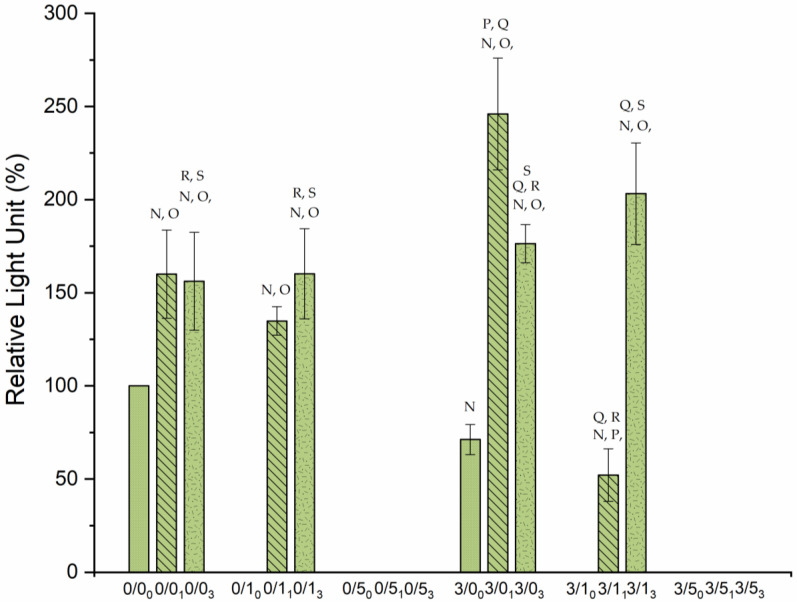
Biofilm viability results created on PLA film in percentage of relative light units. The smooth bars are for non-degraded samples, the diagonally hatched bars are for the samples degraded for one month, and the notched bars are for the samples degraded for 3 months. Statistical difference (*p* < 0.05) is marked with the different letters depending on which sample it is compared with. The letters ^N^ is used to 0/0_0_; ^O^ is used to 3/0_0_; ^P^ is used to 0/0_1_; ^Q^ is used to 0/1_1_; ^R^ is used to 3/0_1_; ^S^ is used to 3/1_1_.

As with the bacterial adhesion experiments, biofilm formation on the PLA surface depended on the three variables under study: Mg, CTAB and degradation time. In this technique, the ATP amount released from bacterial cells was estimated by measuring the relative light units (RLU) to quantify the biofilm created during 24 h so, information from viable microorganisms was extracted. All the data shown in Figure 7 are relativized to the 0/0_0_ sample, which fits 100%.

- Magnesium effect:
  The single addition of magnesium (without any CTAB) to the PLA films resulted in a decrease of biofilm on their surface when samples were not degraded (0/0_0_ vs. 3/0_0_). In the case of degraded samples, Mg made the biofilm significantly increase (*p* < 0.05), especially after 1 month degradation (0/0_1_ vs. 3/0_1_).
  In the case of the addition of magnesium together with CTAB (3/1_0_ or 3/5_0_), Figure 6 shows that no viable biofilm was found.
  Biofilm creation in degraded samples exhibited different behavior depending on the presence of Mg in the sample. In the samples containing 1% (*w*/*w*) CTAB, when films suffered degradation, Mg significantly decreased (*p* < 0.05) biofilm after 1 month (0/1_1_ vs. 3/1_1_) and it practically remained constant after 3 months (0/1_3_ vs. 3/1_3_). In the samples with 5% (*w*/*w*) CTAB, Mg presence was not relevant since biofilm was suppressed at this surfactant concentration.
- CTAB effect
  As already mentioned, the presence of CTAB in non-degraded films, whatever the amount, did not generate viable biofilm. This bactericidal effect was always maintained in 5% (*w*/*w*) regardless of further degradation undergone.
  In samples degraded for 1 month, the addition of 1% (*w*/*w*) CTAB caused a significant reduction (*p* < 0.05) in biofilm formation only in the presence of Mg: from 243 ± 30% RLU (3/0_1_) to 52 ± 14% RLU (3/1_1_).
- Degradation effect
  Degradation time did not affect biofilm formation in the same way for the different samples. A general observation is that degradation leads to an increase in biofilm formation, although this absolute increase is not a function of degradation time. Samples with 5% (*w*/*w*) CTAB, either with or without Mg, did not participate in any degradation trend as they did not form biofilm on their surface. Focusing on the samples with 1% (*w*/*w*) CTAB, it was found that all the samples showed a significant increase (*p* < 0.05) in biofilm formation with increased degradation time, especially with Mg doping. Likewise, samples containing neither CTAB nor Mg exhibited the same affinity for biofilm formation at both degradation times. In contrast, in the films 3/0, the maximum biofilm formation occurred after one month of degradation. In particular, the evolution was from 71 ± 8% RLU to 246 ± 30% RLU after 1 month. These changes were significantly larger for both sample 3/0_0_ and sample 3/0_1_. Specifically, samples subjected to a degradation of 3 months generated a similar biofilm coating on their surfaces, within the experimental error, although the highest average value was for 3/1_3_.
  Comparisons between both degradation times show that degradation processes did not affect the biofilms obtained in the samples without magnesium (0/0_1_ vs. 0/0_3_ and 0/1_1_ vs. 0/1_3_). However, a significant decrease (*p* < 0.05) in biofilm was observed in 3/0 with increased degradation time (about 70 units), and a significant increase (*p* < 0.05) in biofilm was observed in 3/1 with increased degradation time (about 150 units).

## 4. Discussion

The presence of surfactants and metallic particles, such as CTAB and Mg, respectively, changes the surface properties of PLA films [34,41]. In addition to these surface changes, substances embedded in the matrix, that can alter the response of these materials to bacterial colonization and material degradation, is a new variable for modifying the adhesive scenario of any microorganism. The results shown in this work have approximated that possible real situation.

In relation to physical surface properties, degradation for 1 and 3 months for magnesium-free films makes the surfaces less hydrophobic in the cases without or with a low amount of CTAB (Figure 2a). In any case, the change is much more noticeable at short degradation periods (after 1 month); in fact, after 3 months of degradation, the films recover some of the hydrophobicity lost after the first month of degradation. This effect may be related to the fast adsorption of the PBS salts on the surface of the films. 

The presence of CTAB has a singular influence on the hydrophobicity of aged films containing Mg. In these cases, the initial decrease in hydrophobicity associated with degradation is not observed, but the initial release of CTAB, both at 1% (*w*/*w*) and 5% (*w*/*w*), significantly increases the hydrophobicity of the films and, after three months, the hydrophobicity decreases very significantly, making them, interestingly, the least hydrophobic surfaces of all the cases studied. This may be due to the fact that in the 3/1 and 3/5 films, in addition to the release of CTAB, the release of Mg particles must be taken into account, which react with the phosphates present in the PBS, creating a magnesium phosphate (MgP) layer that can act as a barrier for the diffusion of the surfactant. Even so, this layer absorbs water, so a decrease in θ_W_ is observed as time passes.

From an energetic point of view, if significant changes in the surface tension of the samples and/or their components are desired to be achieved, it is necessary to dope the material with Mg and CTAB (Table 1). These changes are especially significant after three months of degradation and always greater in the 3/5 samples. The large increase in surface tension in 3/1_3_ and 3/5_3_ agrees with the lower hydrophobicity of these samples and their extremely low ΔG_SWS_ value.

The compositional characterization of the samples (Figure 3), coupled with the previous thermodynamic characterization of the surfaces, reinforces the idea that the release of CTAB from the surface of the films to the degradation medium occurs mainly during the first weeks of contact with the medium. After 1 month, the surfactant is still released into the medium, but at a slower rate. This could be accounted for by the difficulty in diffusion of CTAB into the medium in the salts adsorbed on the surface of the films and the magnesium phosphate layer (in the case of magnesium-containing films). Additional compositional tests using EDX analysis on the surfaces of the samples degraded for 3 months (data not shown) have revealed that the circular clusters detected in AFM images (Figure 4) are salts from the degradation medium. In the case of magnesium-free films, they are mostly sodium chloride crystals, and in the case of the magnesium-containing samples, they are magnesium phosphates.

Bacterial adhesion and biofilm formation are affected not only by the physical surface properties of the films but also by the chemical activity of the polymeric matrix.

In theory, bacterial adhesion at short times can best be related to the physical surface properties of the material, but its quantification can also be strongly affected by the experimental protocol. In this case, surface washings after the 4 h adhesion process may detach the weakly adhered bacteria and, in the end, what is evaluated is a compromise between adhesion and bacterial retention to the surface.

Hydrophobicity has been a parameter closely related to such adhesion and retention, so that more hydrophobic surfaces have been shown to be more capable of retaining microorganisms [46,47,48]. This relationship is well verified on the three PLA surfaces without additives (0/0, 0/0_1_, 0/0_3_): when plotting the number of adhered bacteria against θ_W_ or ΔG_SWS_, the linear fit shows an R^2^ equal to 0.9985 and 0.9989, respectively (data not shown). The addition of Mg and/or CTAB causes the surfaces to change compositionally and/or topographically, which makes it more difficult to find such a direct relationship between adhesion and hydrophobicity. In these cases, the release of positive ions by Mg and/or CTAB may not only alter the electrical interaction between bacteria and surface but also the viable state of the microorganisms, making the final adhesion behavior less predictable.

Working with another polymer, Sankar et al. [49] showed, in their analysis of polydimethylsiloxane (PDMS) doped with CuO-CTAB and ZnO, a decrease in staphylococcal colonization as the hydrophobicity of the polymer decreased. In that study, the presence of CTAB changed the contact angle from 90° to 73° and adhesion decreased by 87%. In our case, we also observed a decrease in the contact angle of PLA + CTAB films and a significant decrease in bacterial adhesion in all CTAB cases with Mg, probably because the Mg^2+^ cations promote the lower adhesion observed. The justification for this can be found in the smaller size of the Mg^2+^ ions compared to the CTAB molecule: Mg^2+^ ions have a greater capacity to penetrate the electrical double layer of bacteria and/or surfaces, compressing and destabilizing them, similar to what can be caused by divalent Cu^2+^ and Zn^2+^ cations on the surfaces in the study by Sankar et al. For this reason, when CTAB is only found in the PLA matrix (without Mg), the reduction in adhesion is not so evident. In line with these results is the work of Azeredo et al. [50]. These authors used a parallel plate flow chamber to evaluate not only the adhesion of *P. fluorescens*, but also the bacterial retention to the surfaces. The results show that CTAB did not promote significant surface removal in contrast to the anionic surfactant SDS (sodium dodecyl sulfate), which was very effective in detaching bacteria from the surface. Their explanation was based on the physico-chemical surface changes provoked by both surfactants: SDS caused an increase in the absolute negative zeta potential and a decrease in hydrophilicity, which explained its high efficiency in removing bacteria from the surface. However, CTAB promoted a fast cell membrane electrical disorganization [51] due to the attractive forces between positive ends of CTAB and negative bacterial surface charge. 

In any case, the presence of CTAB on the surface of the films causes significant damage to the viability of the attached bacteria. During the fabrication process, in the drying of the films, part of the surfactant is exuded to the surface [34] and this produces a significant initial bactericidal action, as evidenced by samples 0/1_0_, 0/5_0_, 3/1_0_ and 3/5_0_. This implies that at least a concentration similar to or higher than the minimum inhibitory concentration (MIC) (0.02 µg/mL, explicitly determined for the strain under study) is currently being released. For guidance, the estimated accumulated amount of CTAB in solution after one month of degradation is 0.37 µg/mL, 0.74 µg/mL, 6.6 µg/mL and 35 µg/mL for 0/1_1_, 3/1_1_, 0/5_1_ and 3/5_1_, respectively [41].

The interaction of CTAB with the bacterial membrane affects its viability, as do the pH and osmolarity of the medium, starvation, extreme temperatures, oxygen concentration, amount of glucose, bactericidal agents or the bacterial metabolic state itself [52,53,54,55]. In particular, in the study by Sathya et al. [56], the effects of CTAB nanocomposites on the bacterial surface structure were investigated using strains with different surface properties. One interesting conclusion was that the bactericidal efficacy depended on the surface composition of the bacterial surface. CTAB-based surfaces, therefore, were more effective against Gram-positive bacteria, in particular bacteria from the *Staphylococcus* genus, due their thick peptidoglycan layer, which provides sites for effective attachment to CTAB nanocomposites. In contrast, Gram-negative bacteria were less vulnerable to them. 

Another factor to consider is how surface degradation affects adhesion. In the topographical analysis of the films, it was found that degradation alters the initial topography [41], mainly at the highest CTAB concentrations, in our work at 5% (*w*/*w*) CTAB. The deposition of salts, the appearance of holes on some surfaces and the release of active substances from the films constitute a scenario in which practically every surface has a different identity. Nevertheless, if we have to highlight some positive behavior with degradation against infections, it is the decrease in the number of bacteria adhered to the 0/0 control surfaces and the non-viability, regardless of the degradation time, of the bacteria adhered to the 3/5 samples. The other samples with CTAB lose their initial bactericidal action after degradation. Magnesium helps both the dispersion of the CTAB in the polymeric matrix and the subsequent release of the surfactant [14]. Additionally, the high density of regular and structured holes of width 1.9 ± 0.2 µm on 3/5 may favor the diffusion of Mg^2+^ ions and CTAB, while this topography may hinder bacterial attachment-retention, as it is the surface with the lowest number of bacteria bound at three months [37,38,57,58].

The complexity of these systems to describe a single antimicrobial behavior is reflected in the work of Wojcieckhowski et al. [59], who working with a copolymer of methyl methacrylate-ethyl acrylate and styrene-ethyl acrylate with CTAB, observed scattered values in antimicrobial activity after surface degradation. Therefore, more research is needed on the use of CTAB in polymeric films for bactericidal purposes, as the focus so far has been on nanoparticles [60] and emulsions [61].

Biofilm formation on surfaces provides complementary information to adhesion. In adhesion, bacteria are suspended in a physiological buffer where their metabolic activity is suppressed, whereas in biofilms, bacteria are in an active metabolic state, quantified by the amount of ATP produced.

The first relevant aspect in the biofilm formation assays (Figure 5) is the complete suppression of biofilm in those samples where bacteria lost their viability after 4 h of adhesion (Figure 5). In addition to these cases, the 0/5 degraded samples also inhibit biofilm formation, demonstrating that the concentration of CTAB molecules released at these long times are able to inhibit bacterial growth on the material, but do not significantly affect the adhesion behavior of the bacteria in a latent state in PBS.

Also of interest in Figure 6 is the increased biofilm formation on aged surfaces (with the exception of 0/5 and 3/5 where there is no biofilm), irrespective of degradation time. It is likely that bacterial anchorage and subsequent proliferation in nutrient-rich media is favored by salt deposition after degradation and, in some cases, by surface hydrophilization and topographical changes [41].

Focusing on Mg, without CTAB, its effect on biofilm formation is dual: on the one hand, it decreases by 28.8% when the surface is not degraded, but after degradation, Mg surfaces have a higher biofilm formation capacity. Previous work with Mg shows not very conclusive results in this respect. It is probably the combination of the concentration and the way the Mg is released that is responsible for the different information found in the literature [62,63,64]. In particular, our group has shown that concentrations of 10% Mg in PLA films are able to initially reduce biofilm formation after 8 h, due to the local change in pH associated with the very fast release of Mg^2+^ but, after 24 h, the subinhibitory concentrations of Mg^2+^ ions provoke a significant increase in biofilm [18]. 

The literature stresses the need for self-healing materials that can prevent bacterial or fungal infections and this fact is evident in different works, such as the recent one by Du et al. [65]. In this research, a detailed study of the bactericidal activity of a polymeric material (PETU, poly(ether-thioureas)) doped with the antimicrobial compounds PEI (poly(ethylene imine)) and CTAB is carried out. With CTAB, bactericidal effects are achieved at lower concentrations than those used in the present work and mechanisms of action are proposed depending on the type of bacteria used. In particular, for Gram-positive bacteria, CTAB causes destruction of the bacterial surface structures, leading to a significant collapse of the bacteria and leakage of the cytoplasm. However, this bacterial killing does not appear to be cytotoxic in fibroblastic cell assays, and the concentration of CTAB 1% *w*/*w* seems to be the ideal concentration in this polymer for self-healing [65]. 

We are aware that the type of polymer, its manufacturing process and its final geometry are decisive in the amount of active substances released into the environment and, in addition, as we have verified, aging of the surface is a new factor to be taken into account when designing biodegradable materials with self-healing properties.

## 5. Conclusions

This work marks a new step towards the design of self-healing biodegradable materials. The results have shown that the presence of Mg and CTAB changes the surface properties of PLA before and after aging and modifies bacterial colonization on the polymer. Bacterial adhesion and biofilm formation are affected by both the physical properties of the surface and the chemical activity of the polymer matrix.

Changes in hydrophobicity are well correlated with adhesion in the absence of active substances in the matrix. In particular, films with CTAB without degradation are completely bactericidal, and this activity is maintained after degradation in cases of higher concentration. The dispersive action of Mg, in terms of the distribution of CTAB within the PLA matrix, turns the 3/5 sample into a highly surface-structured sample that intensifies its antimicrobial action even after degradation.

## Figures and Tables

**Table 1:** Dispersive (γ^d^) and non-dispersive (γ^nd^) components of the surface tension, the total surface tension of the solid (γ_s_), calculated using the Owens–Wendt–Kaelble (OWK) approach and the free energy of interaction between two surfaces (ΔG_SWS_). All data are given as mean and standard deviation.

Sample	γ^d^ (mJ/m^2^)	γ^nd^ (mJ/m^2^)	γ_s_ (mJ/m^2^)	ΔG_SWS_ (mJ/m^2^)	Sample	γ^d^ (mJ/m^2^)	γ^nd^ (mJ/m^2^)	γ_s_ (mJ/m^2^)	ΔG_SWS_ (mJ/m^2^)
0/0_0_	26 ± 1	0 ± 0	26 ± 1	−94 ± 3	3/0_0_	30 ± 7	1 ± 1	31 ± 8	−84 ± 17
0/0_1_	28 ± 3	3 ± 2	31 ± 4	−70 ± 9	3/0_1_	28 ± 3	4 ± 3	32 ± 6	−68 ± 14
0/0_3_	20 ± 3	3 ± 2	23 ± 5	−80 ± 13	3/0_3_	23 ± 2	6 ± 3	29 ± 5	−66 ± 12
0/1_0_	31 ± 1	0 ± 1	32 ± 1	−84 ± 6	3/1_0_	26 ± 1	4 ± 2	30 ± 2	−70 ± 7
0/1_1_	27 ± 2	6 ± 2	33 ± 4	−63 ± 7	3/1_1_	16 ± 3	3 ± 3	19 ± 5	−83 ± 13
0/1_3_	22 ± 6	7 ± 3	28 ± 9	−65 ± 15	3/1_3_	26 ± 1	18 ± 4	45 ± 5	−36 ± 8
0/5_0_	23 ± 2	9 ± 6	32 ± 8	−58 ± 16	3/5_0_	29 ± 1	13 ± 1	42 ± 1	−43 ± 2
0/5_1_	25 ± 3	10 ± 5	36 ± 8	−53 ± 14	3/5_1_	21 ± 3	10 ± 4	31 ± 7	−58 ± 12
0/5_3_	25 ± 1	8 ± 3	33 ± 4	−59 ± 10	3/5_3_	25 ± 2	33 ± 5	58 ± 6	−17 ± 7

## Data Availability

Data are contained within the article.
